# Impacts of industrial agglomeration on industrial pollutant emissions: Evidence found in the Lanzhou–Xining urban agglomeration in western China

**DOI:** 10.3389/fpubh.2022.1109139

**Published:** 2023-01-11

**Authors:** Zhuo Jia, Qi Chen, Heya Na, Yongchun Yang, Jinyao Zhao

**Affiliations:** Ministry of Education Key Laboratory of Western China's Environmental System, College of Earth and Environmental Sciences, Lanzhou University, Lanzhou, China

**Keywords:** Lanzhou–Xining urban agglomeration, spatial econometric analysis, industrial agglomeration, industrial pollutant emissions, spatial effect

## Abstract

Industrial agglomeration does not only promote economic and social prosperity of urban agglomeration, but also increases industrial pollution, which poses a health risk to the general public. The Lanzhou–Xining urban agglomeration in western China is characterized by industrial agglomeration and serious industrial pollution. Based on the county panel data of the Lanzhou–Xining urban agglomeration in western China from 2010 to 2018, a research of the impacts of industrial agglomeration on industrial pollutant emissions was conducted by using spatial analysis technology and spatial econometric analysis. The results indicate that industrial agglomeration is an important factor leading to an increase in industrial pollutant emissions. In addition, population density, economic level, and industrial structure are also important factors that lead to the increase in industrial pollutant emissions. However, technological level has led to the reduction in industrial pollutant emissions. Furthermore, industrial pollutant emissions are not only affected by the industrial agglomeration, population density, economic level, industrial structure, and technological level of the county but also by those same factors in the surrounding counties, owing to the spatial spillover effect. Joint development of green industries and control of industrial pollutant emissions is an inevitable result for the Lanzhou–Xining urban agglomeration in western China.

## 1. Introduction

Industrial agglomeration is the main spatial organization form of industrialization. Through the mechanisms of scale economy, industrial agglomeration promotes economic competitiveness, optimizes the resource allocation, and stimulates technological innovation, which at the same time has also led to an increase in industrial pollutant emissions (1). The increase in industrial pollutant emissions from China's industrialization not only affects public health but also affects the sustainable development of the economy and society (2). The pollution generated from China's industrial production is more serious than that which is generated in daily life, and preventing and controlling the discharge of industrial pollution have become an important goal of environmental pollution control in China (3).

During the past decades, urban agglomerations have seen rapid industrialization, and rapid economic growth along with sharply increasing industrial pollutant emissions (4). Therefore, the impact of industrial agglomeration on industrial pollutant emissions has received much attention in the research community. However, the results of such research have not always been in agreement, owing to the differences in research areas, methods, and objectives. This indicates that the impact of industrial agglomeration on industrial pollutant emissions is a complex issue (5). For instance, the elimination of polluting industries in the eastern and central provinces of China leads to the transfer of these industries to the western provinces of China (6). By acquiring these polluting industries, urban agglomerations in western China have promoted the level of industrial activity, causing environmental problems such as increasing industrial pollutant emissions (7).

Industrial agglomeration is an inevitable result of industrial development in urban agglomerations. Industrial agglomeration can reduce transaction costs, increase returns on scale, and reduce transportation costs, which together promote expansion of the industrial scale (8). Industrial agglomeration is more prone to rapid expansion of the industrial scale, resulting in growing energy consumption and industrial pollutant emissions (9). Therefore, areas with high levels of industrial agglomeration are also areas of significant industrial pollution. There seems to be a simple logic here: industrial agglomeration leads to an increase in industrial pollutant emissions (10). However, this conclusion seems to be contradictory to traditional agglomeration economic theory, which suggests that spatial agglomeration has economies of scale and spillover effects (11). Therefore, whether due to the spillover effect of pollution reduction technology or to the scale effect of environmental factors, industrial agglomeration has contributed to a reduction in pollutant emissions from industry. However, does industrial agglomeration lead to an increase or decrease in industrial pollutant emissions? The truth needs to be verified. With the presence of rapid industrial growth in urban agglomerations in western China, it is necessary and urgent to analyze the impact of industrial agglomeration on industrial pollutant emissions. Answering the above questions would provide an effective reference for improving the impact of industrial activity on urban agglomerations in western China.

## 2. Literature review

Upon reviewing the researches that have been conducted, we have come to the conclusion that industrial agglomeration has an impact on industrial pollutant emissions through the effects of pollution and self-purification. On the one hand, the pollution effect of industrial agglomeration leads to an increase in industrial pollutant emissions, and industrial agglomeration leads to the expansion of industrial production scale, which increases raw material consumption and industrial pollutant emissions through the scale effect (12). In the process of industrial agglomeration, some enterprises exhibit free-riding behavior in industrial pollution reduction, resulting in severe pollution of the industrial agglomeration area (13). In the process of industrial agglomeration, environmental regulations are regarded as a tool for promoting competition for resources. Local governments usually adopt environmental regulations in a race to the bottom, creating a refuge for pollution and promoting pollution agglomeration (14). Meanwhile, the self-purification effect of industrial agglomeration leads to a reduction in industrial pollutant emissions. The scale effect and spillover effect of industrial agglomeration reduce industrial pollutant emissions by sharing pollution treatment equipment and technology (15). Industrial agglomeration reduces industrial pollutant emissions by building symbiotic and mutually beneficial relationships between industries (16). Through industrial agglomeration, environmental regulations mobilize the enthusiasm of enterprises and, in turn, carry out industrial pollution reduction to curb industrial pollutant emissions (17).

In the process of industrial agglomeration, population density, economic level, technological level, and industrial structure impact industrial pollutant emissions. A thorough investigation was conducted on the impact of industrial pollutant emissions in light of the factors mentioned above.

Industrial agglomeration leads to changes in population size in the area. On the one hand, industrial agglomeration can provide more employment opportunities and effectively improve population density. With the increase in population density, more products are produced to meet the material needs of residents, resulting in an increase in industrial pollutant emissions (18). The population, on the other hand, may migrate geographically due to an increase in industrial pollutant emissions, resulting in a reduction in population density and industrial pollutant emissions (19).

Industrial agglomeration leads to changes in economic level in the area. On the one hand, industrial agglomeration promotes economic prosperity and social development, and such increase in economic level leads to an increase in raw material consumption and industrial pollutant emissions (20). On the other hand, when the economic level reaches a certain level, residents express greater demand for local environmental quality, forcing the government to adopt stricter environmental regulations, increase investment in industrial pollution prevention and control, and reduce industrial pollutant emissions. This is known as the Environmental Kuznets Curve (21).

Industrial agglomeration leads to changes in the technological level of the area. On the one hand, industrial agglomeration can promote technology sharing, matching, and learning by building symbiotic and mutually beneficial relationships between industries. Furthermore, enterprises can effectively reduce industrial pollutant emissions by sharing pollution treatment technologies and equipment (22). On the other hand, some studies have concluded that although technological progress has improved the production efficiency and expanded production scale, it has not made the production process more environmentally friendly. When technological level promotes the improvement of the production efficiency, it leads to an expansion in the scale of production. Although the pollutant emissions of a single product would have decreased, industrial pollutant emissions would have actually increased (23).

Industrial gatherings lead to changes in the industrial structure of the area. On the one hand, industrial agglomeration leads to adjustments in industrial structure, which can result in extension of the industrial chain, improvement in the efficiency of resource utilization, and reduction in the proportion of high-polluting industries in the national economic structure, reducing industrial pollutant emissions (24). On the other hand, industrial pollutant emissions will increase if resource utilization efficiency is ignored during the process of industrial structure adjustment and the proportion of high-polluting industries is increased (25).

According to previous studies, industrial agglomeration leads to an increase in industrial pollutant emissions through the pollution effect. It also effectively reduces industrial pollutant emissions through the self-purification effect. Both effects simultaneously play a role in the process of industrial agglomeration. Industrial agglomeration leads to changes in population size, economic level, technological level, industrial structure, and other factors, and these changes all have impacts on industrial pollutant emissions. The pollution effect and self-purification jointly effect determines the impact of industrial agglomeration on industrial pollutant emissions (26).

The spatial effect of industrial agglomeration affects the spatial distribution of industrial pollutant emissions. If spatial factors are ignored, the results of estimating the impact of industrial agglomeration on industrial pollutant emissions will be biased. An industrial pollutant emission is a derivative of industrial agglomeration which means it exceeds the environment's ability to purify itself. Therefore, due to the spatial spillover effect, industrial pollutant emissions in this region have an impact on industrial pollutant emissions in the surrounding areas (27). An increase in industrial pollutant emissions suggests that this region has lower environmental costs and is more likely to form a pollution refuge, attracting polluting industries to gather through pollution dividends, which affects industrial pollutant emissions in adjacent regions (28). Therefore, it is reasonable to include spatial factors in the analysis of the impact of industrial agglomeration on industrial pollutant emissions.

Therefore, the impact of industrial agglomeration on industrial pollutant emissions is not only a practical problem faced by regional socio-economic development, but also a scientific problem to be discussed in terms of environmental economic geography. Previous research has demonstrated that spatial factors play an indispensable role in the impact industrial agglomeration has on industrial pollutant emissions (29). As there are differences in levels of industrial development and industrial pollutant emissions at the provincial, municipal, and county scales, under the background of differentiated industrial developmental policies and industrial pollution control policies, the impact of industrial agglomeration on industrial pollutant emissions of urban agglomeration can be more accurately described based on county scale data (30). Only by integrating factors such as industrial agglomeration, population density, economic level, technological level, and industrial structure into a unified research framework and analyzing the pollution effect and self-purification effect created by these factors together, can the impacts of industrial agglomeration on industrial pollutant emissions be effectively described.

## 3. Materials and methods

### 3.1. Study area

**Figure 1:**
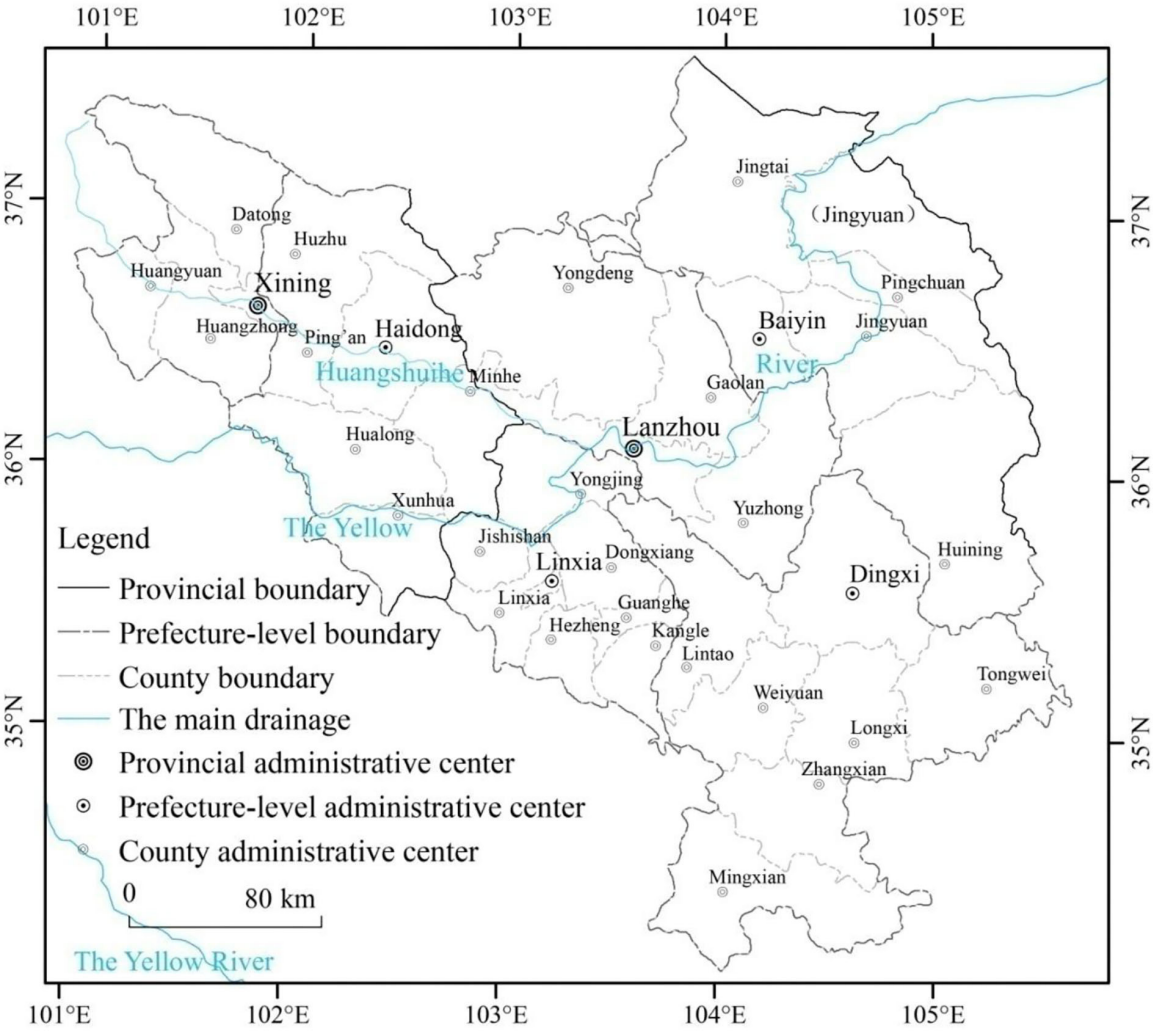
Geographical position and spatial extension of Lanzhou–Xining urban agglomeration in western China.

The Lanzhou–Xining urban agglomeration is an important carrier for economic development and industrial agglomeration, located in the upper reaches of western China. It comprises 41 counties in Lanzhou, Xining, Baiyin, Dingxi, Linxia, and Haidong (Figure 1) (31). Lanzhou–Xining has the typical characteristics of a western China urban agglomeration: (1) Pollution-intensive industries account for a high proportion of the regional industrial structure; (2) Industrial pollutant emissions account for a high proportion of the total regional pollutant emissions. (3) There is a significant contradiction between industrial development and industrial pollution prevention (32). The Lanzhou–Xining urban agglomeration has absolute advantages in the support of pollution-intensive industries, such as the presence of a petrochemical industry, non-ferrous metallurgy, salt chemical industry, building materials and energy supply. Thus, the environmental pressure brought on by pollution-intensive industries is increasingly severe (33). Based on data from 41 counties of the Lanzhou–Xining urban agglomeration in western China from 2010 to 2018, this study examines the spatial characteristics of industrial agglomeration and industrial pollution by using spatial analysis. Spatial factors are introduced into the classic STIRPAT model, and a spatial economic model is constructed to analyze the impact of industrial agglomeration, population density, economic level, technological level, industrial structure, and other factors on industrial pollutant emissions from a spatial perspective, investigating the impact of industrial agglomeration on industrial pollutant emissions in the Lanzhou–Xining urban agglomeration of western China.

### 3.2. Methodology

#### 3.2.1. Calculation of industrial agglomeration

Geographic concentration index is an effective index that considers regional area factors to measure the spatial concentration of industrial activities. The industrial value added represents the outcome of the industrial production activities of industrial enterprises during the reporting period in monetary terms. Thus, industrial agglomeration is defined as the amount of industrial activity per unit area (34). This study calculates the industrial agglomeration level of each county in the Lanzhou–Xining urban agglomeration in western China by using the geographical concentration index. The model is defined as (35).


(1) $$\begin{array}{l} Agg_{it}=\left(x_{it}/\overset{n}{\underset{i=1}{\sum }}x_{it}\right)/\left(TER_{i}/\overset{n}{\underset{i=1}{\sum }}TER_{it}\right)\left(i=1,2,3,\cdots \;,n\right) \end{array}$$

Here, *Agg*_*it*_ is the industrial agglomeration level of county *i* at time *t, x*_*it*_ is the industrial added value of county *i* at time *t*, *TER*_i_ is the area of county *i*, and *n* represents the total number of counties (41) in the Lanzhou–Xining urban agglomeration in western China.

#### 3.2.2. Analysis of spatial correlation

Global Moran's *I* was used to analyze the spatial correlation of industrial pollutant emissions in the Lanzhou–Xining urban agglomeration in western China (36). In case there may be differences in the effects of industrial agglomeration on industrial pollutant emissions of different physical forms, industrial wastewater, industrial SO_2_, and industrial soot are used to represent three different physical forms of industrial pollution. The model is defined as (37).


(2) $$\begin{array}{l} \text{Global Mora}{\text{n}}^{\text{′}}\text{s }\text{I}=\frac{n\sum_{i=1}^{n}\sum_{j=1}^{n}W_{ij}\left(x_{i}-\bar{x}\right)\left(x_{j}-\bar{x}\right)}{\sum_{i=1}^{n}\sum_{j=1}^{n}W_{ij}\sum_{i=1}^{n}{\left(x_{i}-\bar{x}\right)}^{2}} \end{array}$$

Here, *x*_*i*_ and *x*_*j*_ represent the industrial pollutant emissions of county *I* and county *j*, respectively. Variables $\bar{x}$ and*W*_*ij*_ are the mean value and geospatial weight matrix based on Queen's principle, respectively, and *n* is the total number of counties in the Lanzhou–Xining urban agglomeration in western China.

#### 3.2.3. SPIRPAT model

The IPAT model divides the factors affecting environmental change into those involving population, economy, and technology (38). The IPAT model is widely used to analyze the impact of human factors on environmental change because of its concise and representative characteristics. The model is defined as (39).


(3) $$\begin{array}{l} I=P\times A\times T. \end{array}$$

Based on the original variables of the IPAT model, the STIRPAT model is constructed by adding random variables. The standard form of the STIRPAT model is (40).


(4) $$\begin{array}{l} I=\alpha P^{b}A^{c}T^{d}e \end{array}$$

Here, *I* is the environmental impact, *P* is the population size, *A* is the affluence, *T* is the technical level, α is a constant, and *e* is the random error. In addition, *b, c*, and *d* are indexes of population size, affluence, and technical level, respectively. Model (4) can be logarithmically processed to obtain an empirical model (41).


(5) $$\begin{array}{l} \ln\;I=\ln\;\alpha +b\ln\;P+c\ln\;A+d\ln\;T+\ln\;e. \end{array}$$

Here, *b, c*, and *d* are explanatory variable coefficients. The STIRPAT model allows the inclusion of other explanatory variables in the application process (42). Based on the representative studies that have been carried out, industrial agglomeration and industrial structure are added to the empirical form of STIRPAT model, and STIRPAT is updated to the following model (43).


(6) $$\begin{array}{l} \ln POL_{it}=\beta_{0}+\beta_{1}\ln Agg_{it}+\beta_{2}\ln P_{it}+\beta_{3}\ln A_{it}\\ \;+\beta_{4}\ln T_{it}+\beta_{5}\ln G_{it}+\varepsilon_{it} \end{array}$$

Here, *POL*_*it*_ is the industrial pollutant emissions of county *i* at time *t*. Industrial pollutant emissions are expressed by the industrial wastewater, industrial SO_2_, and industrial soot. *Agg*_*it*_ is the level of industrial agglomeration, expressed by the geographical concentration of industrial added value of county *i* at time *t* (44). *P*_*it*_ is the population density, expressed by the population per unit area of county *i* at time *t* (45). *A*_*it*_ is the economic level, expressed by the per capita GDP of county *i* at time *t* (46). *T*_*it*_ is the technical level, expressed by the industrial energy consumption intensity of county *i* at time *t* (47). *G*_*it*_ is the industrial structure and is expressed by the ratio of the added value of the secondary industry in the regional GDP of county *i* at time *t* (48). β_1_, β_2_, β_3_, β_4_, and β_5_ are explanatory variable coefficients. β_0_ is the constant term, and ε_*it*_ is the random error.

#### 3.2.4. Spatial econometric models

It is more effective to solve the problem of spatial dependence by using spatial econometric models, which cannot be handled by linear regression analysis. By adding the spatial term of explanatory variables and the spatial term of explained variables to the STIRPAT model, the spatial impact of industrial agglomeration on industrial pollutant emissions can be examined effectively (49). Based on the improved SPIRTAT model, a spatial econometric model is established. Spatial econometric models mainly include spatial lag panel data model (SLM), spatial error panel data model (SEM), and spatial Durbin panel data model (SDM). This study offers three alternative models (50).

(1) Spatial lag panel data model (SLM):

(7) $$\begin{array}{l} \text{ln}POL_{it}=\rho \sum_{j=1}^{n}W_{ij}\ln POL_{jt}+\beta_{1}\ln Agg_{it}+\beta_{2}\ln P_{it}\\ \;+\beta_{3}\ln A_{it}+\beta_{4}\ln T_{it}+\beta_{5}\ln G_{it}+\mu_{i}+\lambda_{i}+\varepsilon_{it}. \end{array}$$

Here, *POL*_*it*_ is the industrial pollutant emissions of county *i* at time *t*. *Agg*_*it*_, *P*_*it*_, *A*_*it*_, *T*_*it*_, and *G*_*it*_ represent the industrial agglomeration level, population density, economic level, technical level, and industrial structure, respectively, of county *i* at time *t*. Additionally, ρ represents the spatial lag coefficient, and *W*_*ij*_ is the geospatial weight matrix based on Queen's principle. β_1_, β_2_, β_3_, β_4_, and β_5_ are explanatory variable coefficients, μ_*i*_ represents the space fixed effect, λ_*i*_ represents the time fixed effect, and ε_*it*_ is the random error.

(2) Spatial error panel data model (SEM):

(8) $$\begin{array}{l} \text{ ln}POL_{it}=\beta_{0}+\beta_{1}\ln Agg_{it}\text{+}\beta_{2}\ln P_{it}+\beta_{3}\ln A_{it}\\ +\beta_{4}\ln T_{it}+\beta_{5}\ln G_{it}+\mu_{i}+\lambda_{i}+\delta \sum_{j=1}^{n}W_{ij}\phi_{it}+\varepsilon_{it}. \end{array}$$

Here, *POL*_*it*_ is the industrial pollutant emissions of county *i* at time *t*. *Agg*_*it*_, *P*_*it*_, *A*_*it*_, *T*_*it*_, and *G*_*it*_ represent the industrial agglomeration level, population density, economic level, technical level, and industrial structure, respectively, of county *i* at time *t*. β_0_ is the constant term. β_1_, β_2_, β_3_, β_4_, and β_5_ are explanatory variable coefficients, δ is the spatial error coefficient, and *W*_*ij*_ is the geospatial weight matrix based on Queen's principle. *W*_*ij*_φ_*it*_ is the spatial error term, μ_*i*_ is the space fixed effect, λ_*i*_ is the time fixed effect, and ε_*it*_ is the random error.

(3) Spatial Durbin panel data model (SDM):

(9) $$\begin{array}{l} \text{ln}POL_{it}=\rho \sum_{j=1}^{n}W_{ij}\ln POL_{it}+\beta_{1}\ln Agg_{it}+\beta_{2}\ln P_{it}\\ +\;\beta_{3}\ln A_{it}+\beta_{4}\ln T_{it}+\beta_{5}\ln G_{it}+\theta_{1}\sum_{j=1}^{n}W_{ij}\beta_{1}\ln Agg_{it}\\ +\;\theta_{2}\sum_{j=1}^{n}W_{ij}\ln P_{jt}+\theta_{3}\sum_{j=1}^{n}W_{ij}\ln A_{jt}+\theta_{4}\sum_{j=1}^{n}W_{ij}\ln T_{jt}\\ +\;\theta_{5}\sum_{j=1}^{n}W_{ij}\ln G_{jt}+\mu_{i}+\lambda_{i}+\varepsilon_{it}. \end{array}$$

Here, *POL*_*it*_ is the industrial pollutant emissions of county *i* at time *t*. *Agg*_*it*_, *P*_*it*_, *A*_*it*_, *T*_*it*_, and *G*_*it*_ represent the industrial agglomeration level, population density, economic level, technical level, and industrial structure, respectively, of county *i* at time *t*. β_1_, β_2_, β_3_, β_4_, and β_5_ are explanatory variable coefficients. θ_1_, θ_2_, θ_3_, θ_4_, and θ_5_ are spatial autocorrelation coefficients of the explanatory variables, ρ represents the spatial lag coefficient, *W*_*ij*_ is the geospatial weight matrix based on Queen's principle, μ_*i*_ is the space fixed effect, λ_*i*_ is the time fixed effect, and ε_*it*_ is the random error.

### 3.3. Data source

Based on the availability and statistical consistency of the data, this study used the county-based data from the Lanzhou–Xining urban agglomeration in western China from 2010 to 2018 (Table 1). To make the empirical data more consistent with the normal distribution and eliminate heteroscedasticity in the model, the economic and social data, industrial pollutant emission data, and energy consumption data were processed logarithmically before the model estimation. ① Economic and social data. The economic and social data of the 41 counties in the Lanzhou–Xining urban agglomeration from 2010 to 2018, which included the resident population, industrial added value, added value of the secondary industry, and regional GDP, were derived from the 2011–2019 Gansu Development Yearbook, 2011–2019 Qinghai Statistical Yearbook, and Statistical Yearbooks of Lanzhou, Xining, Baiyin, Dingxi, Linxia, and Haidong over the years, adjusting the economic data to the price in 2010, according to the GDP deflator. ② Industrial pollutant emission data and energy consumption data. Data of industrial pollutant emissions and energy consumption from the 41 counties in the Lanzhou–Xining urban agglomeration from 2010 to 2018 were taken from the Environmental Statistical Systems of Lanzhou, Xining, Baiyin, Dingxi, Linxia, and Haidong. ③ Geospatial data. From 2010 to 2018, the administrative divisions of the 41 counties in the Lanzhou–Xining urban agglomeration were slightly adjusted in general. To show the comparison, the geospatial data are based on the 2015 China county administrative boundary data, provided by the resource and environment science and data center of the Chinese Academy of Sciences.

**Table 1:** Definition and explanation of variables.

**Variables**	**Definition**	**Sample size**	**Std.Dev**	**Min**	**Max**
Water	Industrial wastewater emission (10,000 tons)	9^*^41 = 369	4.29	0.20	3,245
SO_2_	Industrial SO_2_ emission (tons)	9^*^41 = 369	8.52	10.00	46,126
SOOT	Industrial SOOT emission (tons)	9^*^41 = 369	4.15	5.00	25,000
*Agg*	Geographical concentration of industrial added value	9^*^41 = 369	10.57	0.02	67.23
*P*	Population density (10,000 people/km^2^)	9^*^41 = 369	3.12	0.41	0.59
*A*	Economic level (10,000 Yuan/person)	9^*^41 = 369	1.22	1.35	7.15
*T*	Industrial energy consumption intensity (standard coal/10,000 Yuan)	9^*^41 = 369	3.15	2.30	10.27
*G*	Percentage secondary industries in GDP (%)	9^*^41 = 369	2.37	0.34	0.78

## 4. Results

### 4.1. Spatial pattern of industrial agglomeration

**Figure 2:**
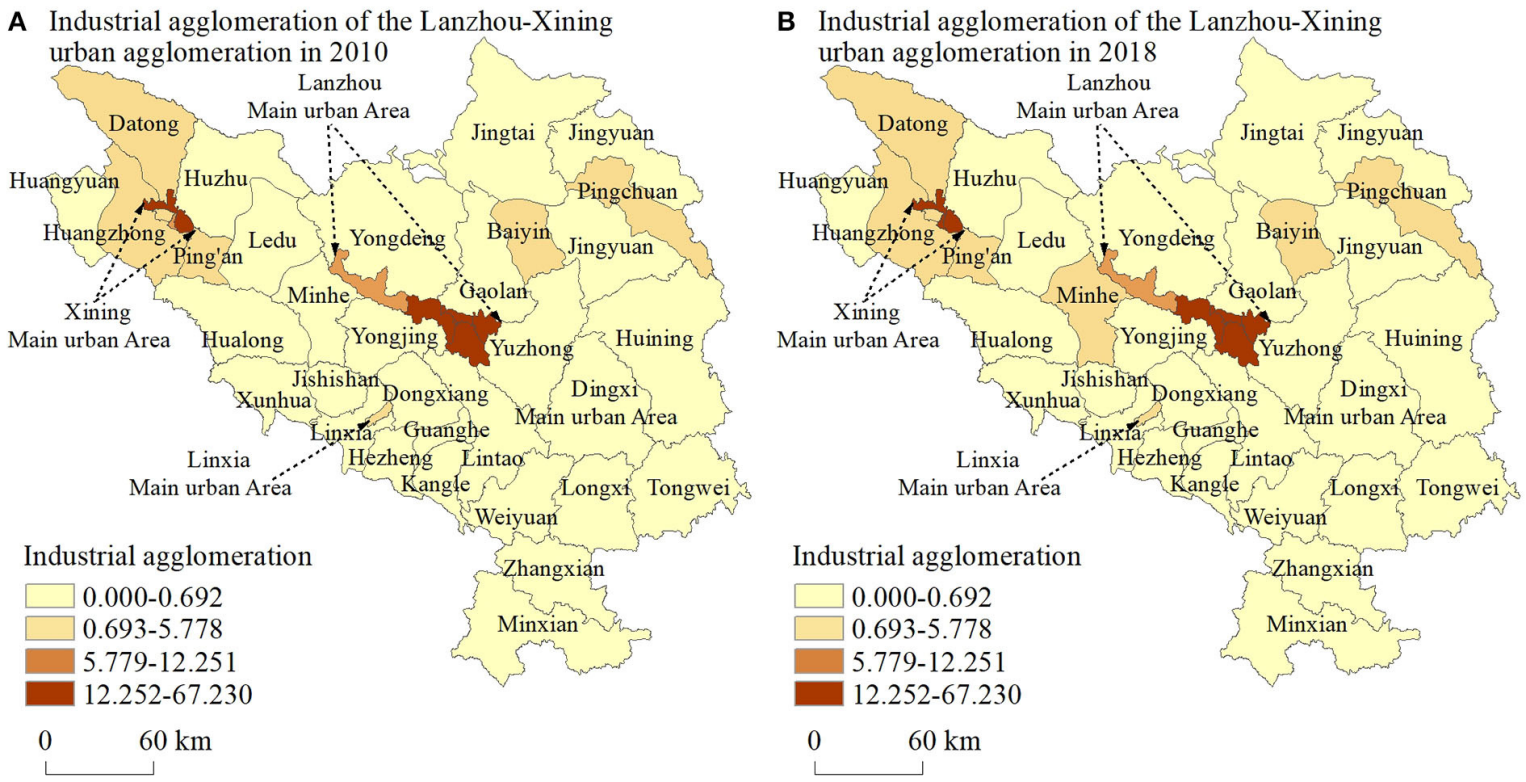
Spatial pattern of industrial agglomeration of Lanzhou–Xining urban agglomeration in western China.

According to Formula 1, the industrial agglomeration of the Lanzhou–Xining urban agglomeration was calculated, and spatial visualization was carried out through ArcGIS10.2 (Figure 2). The spatial pattern of industrial agglomeration of the Lanzhou–Xining urban agglomeration in western China has the following characteristics. ① Spatial imbalance. In the 1950s and 1970s, Lanzhou, Xining, and Baiyin were regarded as important industrial and energy bases in western China and won key construction projects in China. Owing to the inertia of industrial development, Lanzhou, Xining, and Baiyin have become the high-level areas of industrial agglomeration in the Lanzhou–Xining urban agglomeration. Meanwhile, cities with relatively slow industrial development, such as Dingxi, Linxia, and Haidong, have become the low-level areas of industrial agglomeration in the Lanzhou–Xining urban agglomeration. ② Matthew effect. On the one hand, population, capital, technology, and other factors continue to flow from the low-level areas of industrial agglomeration to the high-level areas through the siphon effect. On the other hand, the high-level areas of industrial agglomeration prioritize becoming the geographical space to assume the transferred industries from the eastern and central regions. Consequently, the industrial agglomeration level difference among the counties under the jurisdiction of the urban agglomeration has gradually increased. In 2010, the industrial agglomeration level was between 0.02 and 42.64, while in 2018, the industrial agglomeration level was between 0.03 and 67.23. The ratio of the highest value to the lowest value increased from 1888.15 to 2389.93.

### 4.2. Spatial pattern of industrial pollutant emissions

**Figure 3:**
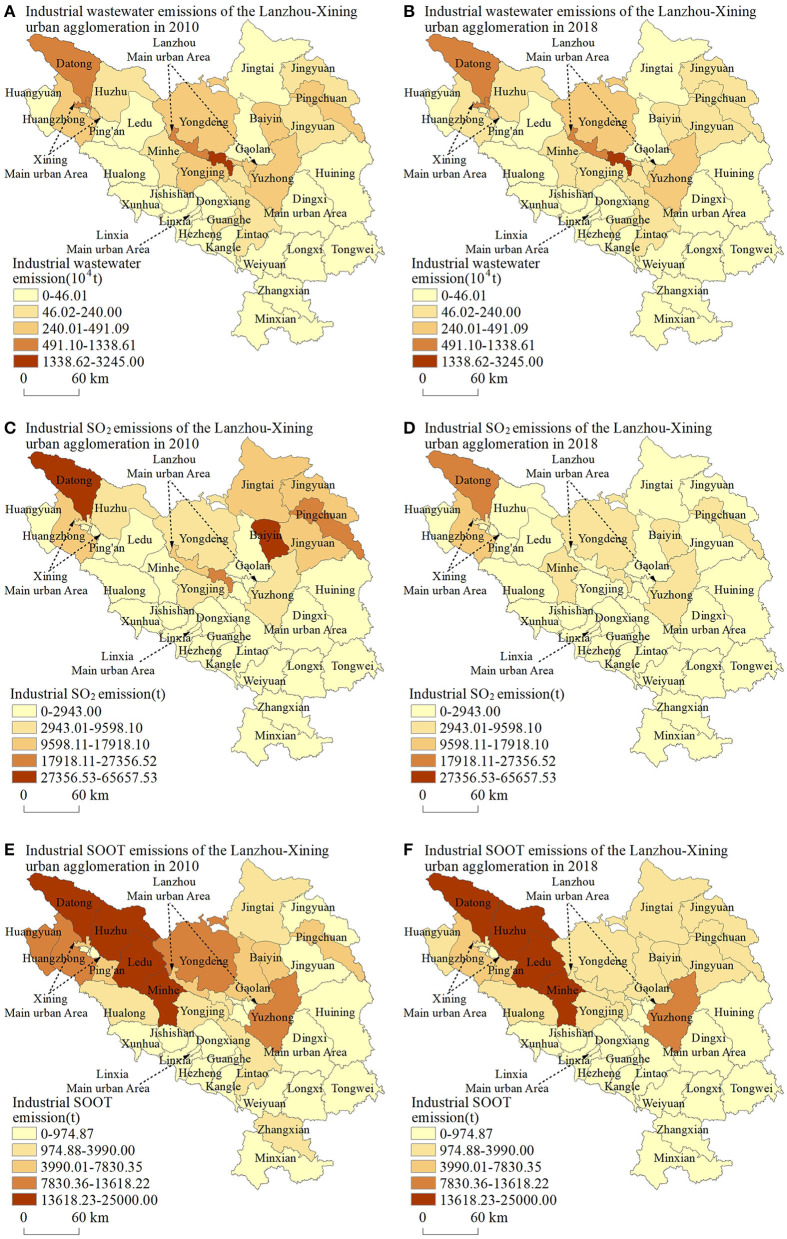
Spatial pattern of industrial pollutant emissions of Lanzhou–Xining urban agglomeration in western China.

ArcGIS10.2 was used to visualize the industrial pollutant emissions of the Lanzhou–Xining urban agglomeration in western China in 2010 and 2018 (Figure 3). The spatial pattern of the industrial pollutant emissions of the Lanzhou–Xining urban agglomeration in western China has the following characteristics. ① Spatial patterns of industrial pollutant emissions and industrial agglomeration have spatial convergence. High-level areas of industrial pollutant emissions are concentrated in Lanzhou, Xining, and Baiyin, whereas low-level areas of industrial pollutant emissions are concentrated in Dingxi, Linxia, and Haidong. ② Industrial pollutant emissions have generally declined. Industrial pollutant emissions have been effectively controlled as a result of the continuous implementation of the concept of ecological civilization and the deepening of pollution prevention and management. From 2010 to 2018, the gap between the high-level areas and low-level areas of industrial pollutant emissions among the 41 counties of the Lanzhou–Xining urban agglomeration in China has been narrowing. In 2010, the county with the highest industrial wastewater emissions had 3,245 × 10^4^ tons of emissions, and the county with the lowest industrial wastewater emissions had 0.2 × 10^4^ tons; meanwhile, in 2018, the county with the highest industrial wastewater emissions had 2,258 × 10^4^ tons of emissions, and the county with the lowest industrial wastewater emissions had 0.2 × 10^4^ tons. In 2010, the county with the highest industrial SO_2_ emissions emitted 65,658 tons, and the county with the lowest industrial SO_2_ emissions emitted 24 tons; in 2018, the county with the highest industrial SO_2_ emissions accounted for 23,000 tons, and the county with the lowest industrial SO_2_ emissions accounted 10 tons. In 2010, the county with the highest industrial soot emissions yielded 25,000 tons, and the county with the lowest industrial soot emissions yielded 7 tons; meanwhile, in 2018, the county with the highest industrial soot emissions yielded 24,477 tons, and the county with the lowest industrial soot emissions yielded 5 tons.

### 4.3. Estimation results of traditional panel data model

This linear regression model is commonly estimated by OLS. Tables 2–4 show the impact of industrial agglomeration on industrial wastewater emissions, industrial SO_2_ emissions, and industrial soot emissions, respectively, by OLS. It can be seen from the estimation results in Tables 2–4 that the regression coefficient of industrial agglomeration is significantly positive when the spatial effect is not considered, indicating that the improvement in industrial agglomeration leads to an increase in industrial pollutant emissions. Meanwhile, the impact of industrial agglomeration on industrial wastewater emissions, industrial SO_2_ emissions, and industrial soot emissions is different. The impact of industrial agglomeration on industrial SO_2_ emissions is the most significant, whereas the impact on industrial wastewater emissions is the weakest. Meanwhile, the impact of industrial agglomeration on industrial soot emissions is at the intermediate level. The regression coefficients of economic level, population density, and industrial structure are all significantly positive, indicating that economic level, population density, and industrial structure have a promoting effect on industrial pollutant emissions. The regression coefficients of the technical level are significantly negative, indicating that the technical level has an inhibitory effect on the industrial pollutant emissions.

**Table 2:** OLS estimation results of industrial agglomeration impact on industrial wastewater emissions.

**Determinants**	**Model (1)**	**Model (2)**	**Model (3)**	**Model (4)**	**Model (5)**
ln *Agg_*it*_*	0.213^**^(3.21)	0.241^**^(3.45)	0.225^*^ (3.09)	0.217^**^(3.07)	0.205^**^(2.95)
ln *P_*it*_*	-	0.119^*^(2.45)	0.203^*^(2.78)	0.117^*^(2.76)	0.122^*^(2.46)
ln *A_*it*_*	-	-	0.175^*^(2.56)	0.154^*^(2.34)	0.134^*^(2.32)
ln *T_*it*_*	-	-	-	−0.107^*^ (2.08)	−0.121^*^(2.03)
ln *G_*it*_*	-	-	-	-	0.251^***^(3.27)
R^2^	0.256	0.267	0.264	0.252	0.283
Obs	369	369	369	369	369

In parentheses the *t*-values are given. ^***^, ^**^, or ^*^ indicates significance at the 1, 5, and 10% levels, respectively.

**Table 3:** OLS estimation results of industrial agglomeration impact on industrial SO_2_ emissions.

**Determinants**	**Model (1)**	**Model (2)**	**Model (3)**	**Model (4)**	**Model (5)**
ln *Agg_*it*_*	0.281^*^ (3.89)	0.254^*^ (3.63)	0.237^*^ (3.32)	0.243^**^ (3.41)	0.261^*^ (3.52)
ln *P_*it*_*	**-**	0.101^*^ (2.02)	0.190^*^ (2.38)	0.182^*^ (2.29)	0.131^*^ (2.17)
ln *A_*it*_*	**-**	-	0.106^*^ (2.26)	0.117^*^ (2.42)	0.115^*^ (2.36)
ln *T_*it*_*	**-**	-	-	−0.119 (2.28)	−0.106^*^ (2.14)
ln *G_*it*_*	**-**	**-**	**-**	-	0.270^**^ (3.89)
R^2^	0.297	0.294	0.301	0.322	0.341
Obs	369	369	369	369	369

In parentheses the *t*-values are given. ^***^, ^**^, or ^*^ indicates significance at the 1, 5, and 10% levels, respectively.

**Table 4:** OLS estimation results of industrial agglomeration impact on industrial soot emissions.

**Determinants**	**Model (1)**	**Model (2)**	**Model (3)**	**Model (4)**	**Model (5)**
ln *Agg_*it*_*	0.203^*^ (2.35)	0.216^*^ (3.10)	0.276^*^ (3.52)	0.217^***^ (3.09)	0.223^*^ (2.65)
ln *P_*it*_*	**-**	0.142^*^ (2.54)	0.174^*^ (2.96)	0.155^*^ (2.94)	0.120^*^ (2.33)
ln *A_*it*_*	**-**	-	0.107^*^ (2.31)	0.138^*^ (2.73)	0.124^*^ (2.51)
ln *T_*it*_*	**-**	-	-	−0.131 (2.11)	−0.141^*^ ((2.36)
ln *G_*it*_*	**-**	**-**	**-**	-	0.171^**^ (2.73)
R^2^	0.224	0.245	0.256	0.278	0.287
Obs	369	369	369	369	369

In parentheses the *t*-values are given. ^***^, ^**^, or ^*^ indicates significance at the 1, 5, and 10% levels, respectively.

### 4.4. Spatial autocorrelation test

Based on GeoDa 1.14, global Moran's *I* was used to analyze the spatial correlation of industrial wastewater emissions, industrial SO_2_ emissions, and industrial soot emissions of the Lanzhou–Xining urban agglomeration in western China. It can be seen from Table 5 that Moran's *I* of industrial wastewater emissions, industrial SO_2_ emissions, and industrial soot emissions from 2010 to 2018 are all positive, and the *p*-value has passed the 5% significance level test. Areas with high levels of industrial pollutant emissions are adjacent to areas with high levels of industrial pollutant emissions, whereas areas with low levels of industrial pollutant emissions are adjacent to areas with low levels of industrial pollutant emissions (51). This means that the industrial wastewater emissions, industrial SO_2_ emissions, and industrial soot emissions all have spatial positive correlation. According to the analysis of global Moran's *I* for industrial wastewater emissions, SO_2_ emissions, and industrial soot emissions, the spatial positive correlation of industrial wastewater emissions is the weakest, and the global Moran's *I* is between 0.101 and 0.151. The spatial positive correlation of industrial soot emissions is the most significant, and the global Moran's *I* is between 0.219 and 0.297. The spatial positive correlation of industrial SO_2_ emission is at the intermediate degree, and the global Moran's *I* is between 0.115 and 0.177. Based on GeoDa 1.14, Global Moran's *I* was used to analyze the spatial correlation of industrial agglomeration level, population density, economic level, technical level, and industrial structure of the Lanzhou–Xining urban agglomeration in western China (52). It can be seen from Table 5 that Moran's *I* of industrial agglomeration level, population density, economic level, technical level, and industrial structure from 2010 to 2018 are all positive, and the *p*-value has passed the 5% significance level test.

**Table 5:** Global Moran's *I* statistics of and industrial pollutant emissions, industrial agglomeration level, population density, economic level, technical level, and industrial structure.

**Years**	**Water**	**SO_2_**	**SOOT**	**Agg**	***P***	**A**	**T**	**G**
2010	0.131^***^	0.156^**^	0.297^***^	0.250^***^	0.269^***^	0.235^***^	0.117^**^	0.175^***^
2011	0.101^**^	0.126^**^	0.223^***^	0.236^***^	0.252^***^	0.257^***^	0.121^**^	0.165^***^
2012	0.120^**^	0.117^**^	0.249^***^	0.247^***^	0.249^***^	0.243^***^	0.125^**^	0.160^***^
2013	0.151^***^	0.132^**^	0.233^***^	0.255^***^	0.243^***^	0.242^***^	0.127^**^	0.121^**^
2014	0.140^***^	0.115^**^	0.273^***^	0.285^***^	0.248^***^	0.267^***^	0.128^**^	0.112^**^
2015	0.146^***^	0.154^***^	0.270^***^	0.275^***^	0.266^***^	0.270^***^	0.114^**^	0.124^**^
2016	0.145^**^	0.177^***^	0.246^***^	0.278^***^	0.267^***^	0.269^***^	0.119^**^	0.157^***^
2017	0.151^***^	0.157^***^	0.224^***^	0.279^***^	0.269^***^	0.271^***^	0.121^**^	0.160^***^
2018	0.143^***^	0.163^***^	0.219^***^	0.281^***^	0.270^***^	0.274^***^	0.125^**^	0.162^***^

^***^, ^**^, or ^*^ indicates significance at the 1, 5, and 10% levels, respectively.

### 4.5. Model selection

As the industrial wastewater emissions, industrial SO_2_ emissions, and industrial soot emissions of the Lanzhou–Xining urban agglomeration in western China are all positively correlated in space, a spatial econometric model should be built when analyzing the impact of industrial agglomeration on the emission types. As far as model selection is concerned, the spatial panel model should be constructed in such a way that it can be further evaluated through comparison with non-spatial panel models. This research utilizes the LM test for the derived spatial panel data (53). If the results of both the LM-ERR and LM-LAG are not statistically significant, the traditional panel model is chosen; if any of them is significant, then the spatial econometric model is utilized to capture the spatiality.

Our LM test results are presented in Table 6. According to the results, LM-ERR and LM-LAG of industrial wastewater emissions are significant at the 5% level. LM-ERR and LM-LAG of industrial SO_2_ emissions are significant at the 1% level. LM-ERR and LM-LAG of industrial soot emissions are significant at the 5% level. As the LM test rejected the original hypothesis, it shows that the spatial effect should be considered when analyzing the impact of industrial agglomeration on industrial wastewater emissions, industrial SO_2_ emissions, and industrial soot emissions. This spatial effect shows that spatial autocorrelation and spatial error correlation coexist in the model. Thus, it is more appropriate to analyze and select the SDM with the dual effects of spatial lag and spatial error autocorrelation at the preliminary stage (54).

**Table 6:** Spatial econometric model selection test.

**Determinants**	**Water**		**SO**_**2**_		**Soot**	
	**Statistics**	***P*-value**	**Statistics**	***P*-value**	**Statistics**	***P*-value**
LM spatial lag	7.362	0.022	8.639	0.005	9.638	0.035
LM spatial error	5.638	0.037	6.397	0.004	5.875	0.040
Wald test spatial lag	45.862	0.001	52.364	0.000	63.214	0.023
Wald test spatial error	37.661	0.007	36.530	0.000	55.638	0.025
LR test spatial lag	32.255	0.003	26.326	0.001	28.351	0.023
LR test spatial error	41.261	0.001	35.612	0.000	33.652	0.032
Hausman test	86.231	0.002	72.585	0.032	82.657	0.017

The Wald test and LR test were used to determine whether SDM would degenerate into SLM or SEM. As shown in Table 6, the Wald test spatial lag, Wald test spatial error, LR test spatial lag, and LR test spatial error of industrial wastewater emissions are all significant at the 1% level. The Wald test spatial lag, Wald test spatial error, LR test spatial lag, and LR test spatial error of industrial SO_2_ emissions are all significant at the 5% level. The Wald test spatial lag, Wald test spatial error, LR test spatial lag, and LR test spatial error of industrial soot emissions are all significant at the 5% level. Since the Wald test and LR test reject the original hypothesis, it indicates that SDM will not degenerate into SLM or SEM. Therefore, the SDM including the spatial lag dependent variable and spatial autocorrelation error term should be used to analyze the impact of industrial agglomeration on industrial wastewater emissions, industrial SO_2_ emissions, and industrial soot emissions (55).

### 4.6. Spatial econometric regression results

The aim of the Hausman test is to determine whether a model with fixed or random effects is more appropriate. The Hausman test results of the impact of industrial agglomeration on industrial wastewater emissions, industrial SO_2_ emissions, and industrial soot emissions are all significant at the 5% level, rejecting the original assumption of random effects (Table 6). It was found by the Hausmann test that the results significantly reject the original hypothesis, suggesting that the fixed effect should be chosen (56). Therefore, this study should choose a fixed model to analyze the impact of industrial agglomeration on industrial wastewater emissions, industrial SO_2_ emissions, and industrial soot emissions. The fixed model includes a pace fixed effect, time fixed effect, and time–space double fixed effect, and the most suitable effect is selected according to R^2^ and log likelihood (57). As is shown in Table 7, the estimation results of the spatial fixed effect, time fixed effect, and time–space double fixed effect, the significance, and significance test of each explanatory variable have not changed significantly, which indicates that the SDM is robust. The R^2^ and log likelihood parameters of SDM with the spatial fixed effect of industrial agglomeration on industrial wastewater emissions are the largest. The R^2^ and log likelihood parameters of SDM with the spatial fixed effect of industrial agglomeration on industrial SO_2_ emissions are the largest, and the R^2^ and Log likelihood parameters of SDM with the spatial fixed effect of industrial agglomeration on industrial soot emissions are the largest. Therefore, the SDM with the spatial fixed effect is used to estimate the impact of industrial agglomeration on industrial wastewater emissions, industrial SO_2_ emissions, and industrial soot emissions (58). According to the estimation results of SDM with fixed spatial effect, the spatial lag coefficients of industrial wastewater emissions, industrial SO_2_ emissions, and industrial soot emissions are 0.202, 0.244, and 0.230, respectively, and they are all significantly positive at the 1% level (Table 7). This indicates that there is a spatial endogenous interaction effect among the explained variables, indicating that industrial wastewater emissions, industrial SO_2_ emissions, and industrial soot emissions in each county are affected by the relevant factors of the county. They are also affected by the discharge of industrial wastewater emissions, industrial SO_2_ emissions, and industrial soot emissions from adjacent counties.

**Table 7:** Estimation results of spatial Durbin model of industrial agglomeration and industrial pollutant emissions.

**Determinants**	**Spatial fixed effects**			**Time fixed effects**			**Spatial–time fixed effects**		
	**Water**	**SO_2_**	**Soot**	**Water**	**SO_2_**	**Soot**	**Water**	**SO_2_**	**Soot**
*W* × *POL_*it*_*	0.202^***^	0.244^***^	0.230^***^	0.211^***^	0.275^***^	0.254^***^	0.217^***^	0.251^***^	0.235^***^
ln *Agg_*it*_*	0.254^***^	0.262^***^	0.233^***^	0.263^***^	0.274^***^	0.251^**^	0.277^***^	0.285^***^	0.254^**^
ln *P_*it*_*	0.178^***^	0.227^***^	0.165^**^	0.172^**^	0.219^**^	0.173^**^	0.145^**^	0.272^**^	0.170^**^
ln *A_*it*_*	0.132^**^	0.145^**^	0.127^**^	0.131^**^	0.178^**^	0.146^**^	0.151^**^	0.162^**^	0.164^**^
ln *T_*it*_*	−0.117^**^	−0.102^**^	−0.132	−0.074^*^	−0.135^**^	−0.147	−0.165^**^	−0.149^**^	−0.158
ln *G_*it*_*	0.204^***^	0.221^***^	0.218^***^	0.242^***^	0.203^***^	0.279^***^	0.254^***^	0.262^***^	0.214^***^
*W* × ln *Agg_*it*_*	0.120^***^	0.112^***^	0.139^***^	0.110^***^	0.182^***^	0.169^***^	0.140^***^	0.182^***^	0.134^***^
*W* × ln *P_*it*_*	0.043^**^	0.057^**^	0.031^**^	0.067^***^	0.097^***^	0.055^***^	0.063^***^	0.089^***^	0.074^***^
*W* × ln *T_*it*_*	0.050^**^	0.082^**^	0.069^**^	0.098^**^	0.054^**^	0.088^**^	0.074^**^	0.056^**^	0.073^**^
*W* × ln T_it_	−0.093^**^	−0.077^**^	0.081	−0.023^**^	−0.047^**^	−0.031	−0.055^**^	−0.065^**^	−0.071
*W* × ln *G_*it*_*	0.107^***^	0.102^***^	0.109^***^	0.101^***^	0.104^***^	0.109^***^	0.093^***^	0.102^***^	0.113^***^
R^2^	0.672	0.638	0.644	0.568	0.611	0.621	0.532	0.661	0.562
Log likelihood	353.974	274.051	310.124	320.342	223.501	265.321	319.102	258.569	270.224

Numbers in the parentheses represent *t*-values. ^***^, ^**^ and ^*^ indicate significance at the 1, 5, and 10% level.

The regression coefficients of industrial agglomeration with industrial wastewater emissions, industrial SO_2_ emissions, and industrial soot emissions are 0.254, 0.262, and 0.233, respectively, and they are all significantly positive at the level of 1%, indicating that the increase in the industrial agglomeration level in the Lanzhou–Xining urban agglomeration will promote industrial wastewater emissions, industrial SO_2_ emissions, and industrial soot emissions. The spatial term coefficients of industrial agglomeration and industrial wastewater emissions, industrial SO_2_ emissions, and industrial soot emissions are 0.120, 0.110, and 0.139 respectively, and they are all significantly positive at the level of 1%. This indicates that the improvement in the industrial agglomeration level of this county will lead to an increase in the industrial production scale, thus increasing the industrial pollutant emissions of this county, as well as the industrial pollutant emissions of neighboring counties through the scale effect. The trans-boundary nature of industrial pollution discharge is demonstrated here.

The regression coefficients of population density, industrial wastewater emissions, industrial SO_2_ emissions, and industrial soot emissions are 0.178 and 0.227, respectively, which are significantly positive at the level of 1%. The regression coefficient between population density and industrial soot emissions is 0.165, which is significantly positive at the 5% level, indicating that the increase in population density can promote an increase in economic vitality and expansion of the industrial scale, thus increasing industrial wastewater emissions, industrial SO_2_ emissions, and industrial soot emissions. The spatial term coefficients of population density on industrial wastewater emissions, industrial SO_2_ emissions, and industrial soot emissions are 0.043, 0.051, and 0.069, respectively, and they are all significantly positive at the 5% level, indicating that the increase in population density in the county will lead to an increase in resource consumption and industrial pollutant emissions, which will lead to an increase in industrial pollutant emissions in the county. The increase in industrial pollution and environmental deterioration in this country will lead to a flow of residents to the neighboring counties through various policies, resulting in an increase in industrial pollution emissions from the neighboring counties.

The regression coefficients between economic level and industrial wastewater emissions, industrial SO_2_ emissions, and industrial soot emissions are 0.132, 0.145, and 0.127, respectively, and are significantly positive at the 5% level, indicating that industrial pollutant emissions have not crossed the peak of Kuznets curve. At this stage, with an increase in the economic level, industrial wastewater emissions, industrial SO_2_ emissions, and industrial soot emissions will increase. The spatial term coefficients of economic level, industrial wastewater emissions, industrial SO_2_ emissions, and industrial soot emissions are 0.050, 0.083, and 0.037 respectively, which are significantly positive at the 5% level, indicating that the improvement in the economic level of this county will lead to an improvement in the industrial pollution discharge of this county, and the significant promotion of economic level of adjacent counties will lead to an improvement in the industrial pollution discharge of this county. The reason is that due to the economic development in this district, the residents will pay more attention to the quality of their living environment, causing the polluting industries to relocate to the adjacent areas.

The regression coefficients of technical level and industrial wastewater emissions, industrial SO_2_ emissions, and industrial soot emissions are −0.117 and −0.102, respectively, which are significantly negative at the 5% level. Although the regression coefficients of technical level and industrial soot emissions are also negative, they fail to pass the significance test, indicating that technical level has “crowding-out effect” on industrial soot emissions and industrial wastewater emissions and can reduce industrial SO_2_ and wastewater emissions. However, the “technology rebound effect” is more common in industrial production. Although the technology level reduces the industrial soot emissions per unit of production, it does not effectively reduce the total industrial soot emissions, which is not conducive to reducing the industrial soot emissions. The spatial term coefficients of the technical level with industrial SO_2_ emissions and industrial wastewater emissions are −0.093 and −0.077, respectively, which are significantly negative at the 5% level, indicating that the improvement of the technical level of the county can reduce the industrial SO_2_ emissions and industrial wastewater emissions of the county, leading to a reduction in industrial SO_2_ emissions and industrial wastewater emissions of adjacent counties through the demonstration effect. The spatial term coefficient of the technical level on industrial soot emissions is negative, but it fails to pass the significance test. The reason for this result may be the special feature of pollutants and the specific regional distribution of industrial activities.

The regression coefficients of industrial structure with industrial wastewater emissions, industrial SO_2_ emissions, and industrial soot emissions are 0.204, 0.221, and 0.218, respectively, and they are all significantly positive at the 1% level, indicating that with the increase in the proportion of the total output value of the secondary industry in the regional GDP, industrial wastewater emissions, industrial SO_2_ emissions, and industrial soot emissions will increase. The spatial term coefficients of industrial structure and industrial wastewater emissions, industrial SO_2_ emissions, and industrial soot emissions are 0.107, 0.102, and 0.109, respectively, which are significantly positive at the 1% level, indicating that the increase in the proportion of the total output value of the secondary industry in the GDP of the county will not only promote improvement of the industrial pollution discharge of the county, but also lead to improvement of the industrial pollution discharge of adjacent counties through structural effects. That is, when the county increases the proportion of the total output value of the secondary industry in the regional GDP, the neighboring county will race to the bottom to avoid becoming disadvantageous in regional competition and then increase the proportion of the total output value of the secondary industry in the regional GDP, resulting in an increase in industrial pollutant emissions. This shows that joint prevention and control are needed in the process of industrial pollution management.

## 5. Discussion

### 5.1. Research contributions

Scholars have different ideas on the relationship between industrial agglomeration and industrial pollutant emissions. However, there is no consensus on the research conclusion. These differences in variant studies come from the differences in time period, sample, and selection of industrial agglomeration indicators in different studies (59). In the initial stage of industrial agglomeration, it usually leads to an increase in industrial pollution emissions, but under the influence of scale effect and technology effect, it will reduce industrial pollution emissions (60). The industrial agglomeration of Lanzhou Xining urban agglomeration is in its initial stage, and this study is of reference significance to other regions or countries.

The possible innovation of this study mainly lies in the following two aspects. First, this study considers the Lanzhou–Xining urban agglomeration as the sample area to explain the impact of industrial agglomeration in western China on industrial pollutant emissions. The Lanzhou–Xining urban agglomeration is the main reason for the increase in industrial pollutant emissions, owing to the expansion of production scale in the process of industrial agglomeration. The Lanzhou–Xining urban agglomeration in western China needs to achieve high-quality economic development by increasing the proportion of technology intensive industries in the national economic structure, as well as upgrade and transform existing traditional industries. Second, the industrial pollutant emissions of the counties under the jurisdiction of the Lanzhou–Xining urban agglomeration in western China are spatially related, which indicates that the spatial effect of industrial pollutant emissions is closely related to the industrial pollutant emission characteristics of neighboring counties. Therefore, when formulating industrial pollution control policies, an environmental pollution control linkage mechanism at the level of urban agglomeration should be established to prevent the formation of a pollution paradise.

The possible significance of this study mainly lies in the following two aspects. First, in this paper, spatial analysis technology and spatial econometric analysis were not only used to analyze the impacts of industrial agglomeration on industrial pollutant emissions, but also used to analyze the impacts of population density, economic level, scientific and technological level and industrial structure on industrial pollutant emission. Second, the industrial pollutant emission characteristics of the counties in the Lanzhou–Xining urban agglomeration in western China are jointly affected by the industrial agglomeration, population density, economic level, technological level, industrial structure, and other factors of the county and neighboring counties. Therefore, when reducing industrial pollutant emissions, the counties of the Lanzhou–Xining urban agglomeration in western China need to consider the influencing factors of the surrounding counties to promote the overall green industrial transformation and quality improvement of the Lanzhou–Xining urban agglomeration in western China. It is concluded that the coordinated development of industry is the fundamental of joint prevention of pollution.

### 5.2. Deficiencies of the study

Owing to the limitations in research methods and data materials, this study may have some research deficiencies in the following three aspects. First, this study mainly focuses on analyzing the impact of industrial agglomeration on the characteristics of industrial pollutant emissions from the data of county scale industrial pollutant emissions of the Lanzhou–Xining urban agglomeration in western China. However, it fails to effectively dig deeper into the enterprise and industry level to analyze the difference in the impact of industrial agglomeration on industrial pollutant emissions from different industries. This will affect the comprehensiveness of the analysis of the impact of industrial agglomeration on industrial pollutant emissions. Second, owing to the change in the statistical caliber of industrial pollutant emission data, this study focuses on analyzing the impact of industrial agglomeration on industrial pollutant emission characteristics of the Lanzhou–Xining urban agglomeration in western China through the data from 2010 to 2018; thus, there is a lack of long-term and continuous tracking data. Third, the synergy and combination effects among industrial agglomeration, population density, economic level, technological level, and industrial structure have not been analyzed. Meanwhile, the interaction effects of the reverse effects of industrial pollutant emissions on industrial agglomeration have not been deeply analyzed. The above deficiencies will also be the areas and directions for further research.

## 6. Conclusion

The impact of industrial agglomeration on industrial pollutant emissions of the Lanzhou–Xining urban agglomeration in western China is analyzed by using spatial analysis and spatial econometric models. The main conclusions of this study are as follows.

Industrial agglomeration is an important factor leading to the increase in industrial pollutant emissions. Adhering to the environmental bottom line of economic development and promoting green industrial transformation are the primary ways for the Lanzhou–Xining urban agglomeration in western China to achieve high-quality development. Both industrial agglomeration and industrial pollutant emissions have spatial effects, and industrial agglomeration and industrial pollutant emissions of adjacent counties are closely related. Coordinated industrial development is the basis of comprehensive pollution control. Without the common industrial improvement and efficiency, there will be no long-term joint management and control of pollution. It is inevitable that the Lanzhou–Xining urban agglomeration in western China will form a spatial synergy of industrial co-construction and pollution control.

As industrial wastewater emissions, industrial SO_2_ emissions, and industrial soot emissions are different types of pollutants, the spatial patterns are significantly different, leading to significant differences in the main influencing factors of the different pollutant emissions. Industrial pollution discharge is not only affected by the population density, economic level, technological level, industrial agglomeration, industrial structure, and other factors of the county but is also affected by the industrial pollution agglomeration and influencing factors of its neighboring counties. Social and economic factors play a role in industrial pollutant emissions of this county and neighboring counties through spatial effect, and different social and economic factors have different spatial effects.

Industrial pollutant emissions of the Lanzhou–Xining urban agglomeration in western China have spatial positive correlation and spatial spillover. Therefore, it is necessary to take full advantage of the coordination among the counties of the urban agglomeration, pay attention to joint prevention and control, co-construction and common governance, and form a joint force space-wise. Industrial pollutant emissions are a derivative problem in the process of industrial agglomeration development during urban agglomeration; thus, it is necessary to control industrial pollution agglomeration through the coordination of government power, enterprise power, market power, and social power. Industrial pollutant emissions are both an environmental problem and a developmental problem. Therefore, on the premise of maintaining economic development, we should promote industrial structure adjustment and industrial chain extension to improve the quality and efficiency of industry. Meanwhile, we need to optimize the energy structure and technology level as well as to promote and demonstrate emission reduction technologies, improving the overall technological contribution for the pollution reduction. It is concluded that the coordinated development of industry is the fundamental of joint prevention of pollution. It is an inevitable choice to construct a spatial synergy of industrial co-construction and pollution co-governance for the ecological protection and high-quality development of urban agglomeration.

## Data availability statement

The original contributions presented in the study are included in the article/supplementary material, further inquiries can be directed to the corresponding author.

## Author contributions

ZJ for conceptualization, methodology, software, validation, formal analysis, writing, and original draft preparation. QC and YY for investigation, resources, and data curation. HN and JZ for writing—review and editing, supervision, and project administration. All authors have read and agreed to the published version of the manuscript.

## References

[1] Shen N Peng H. Can industrial agglomeration achieve the emission-reduction effect? Socioecon Plann Sci. (2020) 75:100867. 10.1016/j.seps.2020.100867

[2] Zhao H Cao XX Ma T. A spatial econometric empirical research on the impact of industrial agglomeration on haze pollution in China. Air Qual Atmos Health. (2020) 13:1305–12. 10.1007/s11869-020-00884-w31267389

[3] Chen CF Sun YW Lan QX Jiang F. Impacts of industrial agglomeration on pollution and ecological efficiency-a spatial econometric analysis based on a big panel dataset of China's 259 cities. J Clean Prod. (2020) 258:120721. 10.1016/j.jclepro.2020.120721

[4] Liang LW Wang ZB Li JX. The effect of urbanization on environmental pollution in rapidly developing urban agglomerations. J Clean Prod. (2019) 237:117649. 10.1016/j.jclepro.2019.117649

[5] Han X Dou JM Tang CH. Polycentricity, agglomeration, and industrial air pollution in the Chinese city-regions. Front Environ Sci. (2022) 10:879395. 10.3389/fenvs.2022.879395

[6] Liu SX Zhu YM Du KQ. The impact of industrial agglomeration on industrial pollutant emission: evidence from China under new normal. Clean Technol Environ Policy. (2017) 19:2327–34. 10.1007/s10098-017-1407-0

[7] Li XH Xu YY Yao X. Effects of industrial agglomeration on haze pollution: a Chinese city-level study. Energy Policy. (2021) 148:111928. 10.1016/j.enpol.2020.111928

[8] Hilber CAL Voicu I. Agglomeration economies and the location of foreign direct investment: empirical evidence from Romania. Reg Stud. (2010) 44:355–71. 10.1080/00343400902783230

[9] Chen DK Chen SY Jin H. Industrial agglomeration and COsb2 emissions: evidence from 187 Chinese prefecture-level cities over 2005–2013. J Clean Prod. (2018) 172:993–1003. 10.1016/j.jclepro.2017.10.068

[10] Sun PY Yuan Y. Industrial agglomeration and environmental degradation: empirical evidence in Chinese cities. Pacific Econ Rev. (2015) 20:544–68. 10.1111/1468-0106.12101

[11] Bartz S Kelly DL. Economic growth and the environment: theory and facts. Resour Energy Econ. (2008) 30:115–49. 10.1016/j.reseneeco.2007.06.001

[12] Olli T Jari K. Economic growth, pollution, and renewable resources. J Environ Econ Manage. (1993) 24:101–18. 10.1006/jeem.1993.1007

[13] Wagner UJ Timmins CD. Agglomeration effects in foreign direct investment and the pollution haven hypothesis. Environ Resour Econ. (2009) 43:231–56. 10.1007/s10640-008-9236-6

[14] Fredriksson PG List JA Millimet DL. Bureaucratic corruption, environmental policy and inbound us USFDI: theory and evidence. J Public Econ. (2003) 87:1407–30. 10.1016/S0047-2727(02)00016-6

[15] Shangguan XM Hashmi SM Hu HY Wong W-K. Tax competition, environmental regulation and high-quality economic development: an empirical test based on spatial Durbin model. Front Public Health. (2022) 10:982159 10.3389/fpubh.2022.982159 36388326 PMC9650063

[16] Zhuang RL Mi KA Feng ZW. Industrial co-agglomeration and air pollution reduction: an empirical evidence based on provincial panel data. Int J Environ Res Public Health. (2021) 18:12097. 10.3390/ijerph182212097 34831854 PMC8622165

[17] Dong BM Gong J Zhao X. FDI and environmental regulation: pollution haven or a race to the top. J Regulat Econ. (2012) 41:216–37. 10.1007/s11149-011-9162-3

[18] Liu H Cui W Zhang M. Exploring the causal relationship between urbanization and air pollution: evidence from China. Sustain Cities Soc. (2022) 80:103783. 10.1016/j.scs.2022.103783

[19] Deschenes O Greenstone M Shapiro JS. Defensive investments and the demand for air quality: evidence from the NOx budget program. Am Econ Rev. (2017) 107:2958–89. 10.1257/aer.20131002

[20] He CF Huang ZJ Ye XY. Spatial heterogeneity of economic development and industrial pollution in urban China. Stoch Environ Res Risk Assess. (2014) 28:767–81. 10.1007/s00477-013-0736-8

[21] Stern DI. Environmental Kuznets curve. Encyclopedia Energy. (2004) 22:517–25. 10.1016/B0-12-176480-X/00454-X

[22] Han CY Gu ZL Yang HX. Investigate the effects of industrial agglomeration on nitrogen dioxide pollution using spatial panel Durbin and panel threshold models. Front Environ Sci. (2022) 10:844479. 10.3389/fenvs.2022.844479

[23] Vivanco DF van der Voet E. The rebound effect through industrial ecology's eyes: A review of LCA-based studies. Int J Life Cycle Assess. (2014) 19:1933–47. 10.1007/s11367-014-0802-6

[24] Llop M. Economic structured and pollution intensity within the environmental input-output framework. Energy Policy. (2007) 35:3410–7. 10.1016/j.enpol.2006.12.015

[25] He J. Pollution haven hypothesis and environmental impacts of foreign direct investment: the case of industrial emission of sulfur dioxide in Chinese provinces. Ecol Econ. (2006) 60:228–45. 10.1016/j.ecolecon.2005.12.008

[26] Jia Z Chu X Li CX Chen XP. Environmental effects of industrial agglomeration based on the bibliometrical analysis of cnki database. Ecol Environ Sci. (2018) 27:2367–76.

[27] Wei G Zhang Z Ouyang X Shen Y Jiang S Liu B He BJ. Delineating the spatial-temporal variation of air pollution with urbanization in the Belt and Road Initiative area. Environ Impact Assess Rev. (2021) 91:106646. 10.1016/j.eiar.2021.106646

[28] Wu JW Wei YD Chen W Yuan F. Environmental regulations and redistribution of polluting ndustries in transitional China: understanding regional and industrial differences. J Clean Prod. (2019) 206:142–55. 10.1016/j.jclepro.2018.09.042

[29] Kelejian HH Prucha IR. Estimation of simultaneous systems of spatially interrelated cross sectional equations. J Econom. (2004) 118:27–50. 10.1016/S0304-4076(03)00133-7

[30] Zhou K Yin Y Li H Shen YM. Driving factors and spatiotemporal effects of environmental stress in urban agglomeration: evidence from the Beijing–Tianjin–Hebei region of China. J Geograph Sci. (2021) 31:91–110. 10.1007/s11442-021-1834-z

[31] Jia Z Qiang WL Wang YJ Li EL Chen XP. The spatial characteristics and spatial effect of industrial pollution agglomeration in Lanzhou–Xining urban agglomeration. Econ Geography. (2020) 40:68–75. 10.15957/j.cnki.jjdl.2020.01.00832986700

[32] Jia Z Zhao JY Yang YC Chen XP. Spatial pattern and spatial convergence of environmental regulation efficiency of Lanzhou–Xining urban agglomeration in the Yellow River Basin. Scientia Geographica Sinica. (2022) 42:568–78. 10.13249/j.cnki.sgs.2022.04.002

[33] Jia Z Yang YC Zhao JY Chen XP. The spatial correlation and interaction between industrial agglomeration and pollution agglomeration of Lanzhou–Xining urban agglomeration in the Yellow River Basin. Geogr Res. (2021) 40:2897–913. 10.11821/dlyj020201097

[34] Ramos-Meza CS Jain V Imran M Chawla C Sriyanto S Khan A. Sustainable growth strategy promoting green innovation processes, mass production, and climate change adaptation: a winwin situation. Front Environ Sci. (2022) 10:1059975. 10.3389/fenvs.2022.1059975

[35] Jia Z Chen XP. Research on country economic aggregation pattern and spatial spillover in Lanzhou Xining urban aggregation in western China. J Lanzhou Univ Social Sci. (2019) 47:144–51. 10.13885/j.issn.1000-2804.2019.02.018

[36] Getis A. A history of the concept of spatial autocorrelation: a geographer's perspective. Geogr Anal. (2010) 40:297–309. 10.1111/j.1538-4632.2008.00727.x

[37] Lee J Li SW. Extending Moran's index for measuring spatiotemporal clustering of geographic events. Geogr Anal. (2017) 49:36–57. 10.1111/gean.12106

[38] Ehrlich PR Holdren JP. Impact of population growth. Science. (1971) 171:1212–1217. 10.1126/science.171.3977.1212 5545198

[39] Liddle B. Population, affluence, and environmental impact across development: evidence from panel cointegration modeling. Environ Model Softw. (2013) 40:255–66. 10.1016/j.envsoft.2012.10.002

[40] Dietz T Rosa EA. Rethinking the environmental impacts of population, affluence and technology. Hum Ecol Rev. (1994) 1:277–300.

[41] York R Rosa EA Dietz T A. rift in modernity? Assessing the anthropogenic sources of global climate change with the STIRPAT model. Int J Sociol Social Policy. (2003) 3:31–51. 10.1108/01443330310790291

[42] Zilio M Recalde M. GDP and environment pressure: the role of energy in Latin America and the Caribbean. Energy Policy. (2011) 39:7941–9. 10.1016/j.enpol.2011.09.049

[43] Kirk RW Bolstad PV Manson SM. Spatio-temporal trend analysis of long-term development patterns (1900–2030) in a Southern Appalachian County. Landscape Urban Plan. (2012) 104:47–58. 10.1016/j.landurbplan.2011.09.008

[44] Dong F Wang Y Zheng L Li JY Xie SX. Can industrial agglomeration promote pollution agglomeration? Evidence from China. J Cleaner Prod. (2019) 246:118960. 10.1016/j.jclepro.2019.118960

[45] Wang GX Shi XW. Cui HY, Jiao J. Impacts of migration on urban environmental pollutant emissions in China: a comparative perspective. J Geograph Sci. (2020) 30:45–58. 10.1007/s11769-020-1096-1

[46] Liu HJ Pei YF. Economic development and China's urban haze pollution: based on spatial correlation networks. Chinese J Urban Environ Stud. (2019) 7:6–37.

[47] Wang ZB Liang LW. Wang XJ. Spatiotemporal evolution of PM25 concentrations in urban agglomerations of China. J Geograph Sci. (2021) 31:878–98. 10.1007/s11442-021-1876-2

[48] Chen YF Xu Y Wang FY. Air pollution effects of industrial transformation in the Yangtze river delta from the perspective of spatial spillover. J Geograph Sci. (2022) 32:156–76. 10.1007/s11442-021-1929-6

[49] Lu W Tam VW Du L Chen H. Impact of industrial agglomeration on haze pollution: new evidence from Bohai Sea economic region in China. J Clean Prod. (2021) 280:124414. 10.1016/j.jclepro.2020.124414

[50] Hao Y. Liu YM. The influential factors of urban PM 25 concentrations in China: a spatial econometric analysis. J Cleaner Prod. (2016) 112:1443–53. 10.1016/j.jclepro.2015.05.005

[51] Zhang D Zhou C He BJ. Spatial and temporal heterogeneity of urban land area and PM2.5 concentration in China. Urban Climate. (2022) 45:101268. 10.1016/j.uclim.2022.101268

[52] Liu H Fang C Zhang X Wang Z Bao C Li F. The effect of natural and anthropogenic factors on haze pollution in Chinese cities: a spatial econometrics approach. J Cleaner Prod. (2017) 165:323–33. 10.1016/j.jclepro.2017.07.127

[53] Elhorst JP. Specification and estimation of spatial panel data models. Int Reg Sci Rev. (2003) 26:244–68. 10.1177/0160017603253791

[54] Zhu L Gan QM Liu Y Yan ZJ. The impact of foreign direct investment on SOsb2 emissions in the Beijing–Tianjin–Hebei region: a spatial econometric analysis. J Clean Prod. (2017) 166:189–96. 10.1016/j.jclepro.2017.08.032

[55] Hao Y Liu YM Weng JH Gao YX. Does the environmental Kuznets curve for coal consumption in China exist? New evidence from spatial econometric analysis. Energy. (2016) 114:1214–23. 10.1016/j.energy.2016.08.075

[56] Li JBA Huang XJ Chuai XW Yang H. The impact of land urbanization on carbon dioxide emissions in the Yangtze river delta, China: a multiscale perspective. Cities. (2021) 116:103275. 10.1016/j.cities.2021.103275

[57] Tsuzuki Y. Relationships between pollutant discharge and water quality in the rivers from “better” to “worse” water quality. Ecol Indic. (2015) 52:256–69. 10.1016/j.ecolind.2014.12.001

[58] Wei ZY Li JM Wang ZY Zhou AQ Li MH. County carbon emissions in the Yangtze River Delta region: spatial layout, dynamic evolution and spatial spillover effects. Front Environ Sci. (2022) 10:977198. 10.3389/fenvs.2022.977198

[59] Liu H Wang C Zhang M Wang S. Evaluating the effects of air pollution control policies in China using a difference-in-differences approach. Sci Total Environ. (2022) 845:157333. 10.1016/j.scitotenv.2022.157333 35842143

[60] Li S J Sun B Hou D X Jin WJ Ji Y. Does industrial agglomeration or foreign direct investment matter for environment pollution of public health? Evidence from China. Front Public Health. (2021) 9:711033. 10.3389/fpubh.2021.711033 34490192 PMC8416622

